# Dr. C. Pereira

**Published:** 1911-04-08

**Authors:** 


The sudden death of Dr. C. Pereira, house surgeon at the Kidderminster Infirmary, who was found dead in his room, has caused regret. He had been at the infirmary about eight months, and previously was a house surgeon at the General Hospital, Birmingham. He was a B.A. of Oxford and L.M.S.S.A., and only qualified in 1910.

